# Labeled dataset of Sentinel-1 SAR imagery Despeckled with multitemporal fusions

**DOI:** 10.1016/j.dib.2026.112999

**Published:** 2026-06-22

**Authors:** Jean Pierre Díaz-Paz, Ahmed Alejandro Cardona-Mesa, Paula Andrea Muñoz-Uribe, Rubén Darío Vásquez-Salazar, Santiago Zapata-Vargas

**Affiliations:** a Faculty of Engineering, Politécnico Colombiano Jaime Isaza Cadavid, Medellín 050022, Colombia; b Faculty of Engineering, Universidad Militar Nueva Granada, Cajicá 250247, Colombia; c Faculty of Sciences and Humanities, Institución Universitaria Digital de Antioquia, Medellín 050012, Colombia

**Keywords:** Synthetic aperture radar, Speckle, Supervised learning, Multitemporal averaging, Google earth engine

## Abstract

This paper presents a labeled multitemporal dataset designed to support supervised despeckling approaches for Sentinel-1 synthetic aperture radar (SAR) imagery. The dataset consists of 512×512-pixel SAR regions acquired in Interferometric Wide Swath (IW) mode (GRD-HD product) for both VV and VH polarizations, paired with corresponding speckle-reduced reference images generated by multitemporal averaging. For each location, ground-truth images were constructed from temporal stacks of 5, 10, 15, 20, and 25 acquisitions, enabling controlled analysis of speckle attenuation as a function of the number of fused scenes. The data were collected globally using an automated workflow implemented in Python and Google Earth Engine, with random spatial sampling and quality filtering based on polarization-specific backscatter thresholds. In addition to the SAR imagery, the dataset includes ESA WorldCover v200 land cover maps and a metadata file containing acquisition parameters and geographic information. Baseline quantitative metrics, including Equivalent Number of Looks (ENL), Mean Squared Error (MSE), Structural Similarity Index (SSIM), and Peak Signal-to-Noise Ratio (PSNR), are provided for representative samples to facilitate benchmarking. The proposed dataset provides a structured, reproducible resource for training and evaluating machine learning and deep learning models for speckle noise reduction in SAR data.

Specifications Table

**Table:** 

Subject	*Earth-Surface Processes, Applied Machine Learning, Global and Planetary Change*
Specific subject area	*Applied remote sensing, Synthetic Aperture Radar (SAR) imagery, and optical imagery*
Data format	*Filtered and processed images in GeoTIFF (.tif) format.*
Type of data	*Folders of Images*
Data collection	*Sentinel-1 SAR imagery was collected in Interferometric wide swath mode, incorporating both VV and VH polarizations, and both ascending and descending orbit directions. The data collection was performed globally using an automated Python workflow via Google Earth Engine based on a stochastic spatial sampling approach. To construct the speckle-reduced ground-truth reference targets, temporal subsets consisting of 5, 10, 15, 20, and 25 consecutive scenes were compiled and processed via multitemporal averaging. Each individual image was cropped to a standardized spatial dimension of 512×512 pixels. Additionally, corresponding land-cover reference layers were systematically extracted for each sampled bounding box using the ESA WorldCover v200 global product.*
Data source location	*The remote sensing data were programmatically accessed and compiled using Google Earth Engine* (https://code.earthengine.google.com/)*, sourcing from the European Space Agency (ESA) Sentinel-1 Copernicus collection* (https://browser.dataspace.copernicus.eu/) *and the ESA WorldCover v200 database* (https://developers.google.com/earth-engine/datasets/catalog/ESA_WorldCover_v200), *using randomized geographic coordinates.*
Data accessibility	Repository name: Mendeley Data Data identification number: DOI: 10.17632/6nzd6x25vw.1 Direct URL to data: https://data.mendeley.com/datasets/6nzd6x25vw/1 Instructions for accessing these data: Go to the URL, navigate to the root folder and its subfolder, and download them. Consider the dataset description.
Related research article	Labeled dataset for training despeckling filters for SAR imagery [1]

## 1 Value of the Data

• The dataset consists of paired images with a pixel resolution of 512×512, comprising both noisy inputs and their corresponding ground-truth references. Different numbers of images (5, 10, 15, 20, and 25) were used with a multitemporal fusion to obtain these ground-truth images, generating several qualities that the researchers can choose according to their specific needs, especially the equivalent number of looks, which describes the level of speckle remaining in these images.
• Such data pairs are particularly suitable for the supervised training of despeckling algorithms. When the dataset includes high-fidelity ground-truth annotations, it enables the development of highly effective despeckling models, enhancing their capacity to generalize and achieve a robust performance.
• The dataset was constructed from real Synthetic Aperture Radar (SAR) imagery acquired by the Sentinel-1 mission, representing a departure from the conventional methodology typically employed in the design and evaluation of speckle filtering techniques. Traditionally, speckle noise is synthetically generated by applying a Gamma-distributed multiplicative model to optical images, which may not fully capture the statistical and structural properties of genuine SAR data. The radar sensor originally generated the speckle present in this dataset through the coherent interference of electromagnetic waves reflected from multiple scatterers.
• The dataset comprises >500 images for each polarization and flight direction, which is sufficient for researchers and developers to split into training and validation sets, allowing them to training machine learning, deep learning, or even generative artificial intelligence models. Nonetheless, the dataset can be further augmented by integrating additional sources, including synthetically generated data, to expand its diversity, facilitate comparative analyses, and potentially improve the generalizability of the resulting models.
• In contrast to most datasets reported in the literature, the present dataset was generated using a novel methodology designed to enhance variability and representativeness. This approach incorporates the random selection of both spatial locations and acquisition dates, ensuring that the data encompass a wide range of geographic conditions, seasonal variations, and imaging scenarios. By introducing this level of diversity, the dataset captures a broader spectrum of speckle patterns and textural characteristics inherent to SAR imagery, which are often rare in conventionally constructed datasets. Consequently, this methodological innovation not only improves the robustness and generalization capabilities of despeckling models trained on the dataset, but also provides a more reliable basis for comparative analyses and benchmarking within the scientific community.

## 2 Background

Recent benchmark initiatives have significantly advanced the availability of labeled radar data. For instance, the OpenEarthMap-SAR dataset [2] was introduced as a benchmark designed for global high-resolution land cover mapping, comprising 1.5 million segments extracted from 5033 aerial and satellite images of 1024×1024 pixels across 35 regions in Japan, France, and the USA. Although OpenEarthMap-SAR provides sub-meter ground sampling distances (0.15–0.5 m) and utilizes a combination of partially manual annotations and fully pseudo 8-class land cover labels to train classification models, its primary focus remains on semantic segmentation rather than the radiometric restoration of the radar signal. The InSAR-DLPU dataset [3], which was introduced as the first public benchmark specifically engineered for DL-based phase unwrapping tasks. This repository comprises 31,100 paired patches of wrapped and absolute phases, synthetically generated from the complete 30-m resolution NASA Shuttle Radar Topography Mission (SRTM) digital elevation model (DEM) database covering China. By embedding a wide richness of topographic features and varying noise levels, InSAR-DLPU aims to systematically enhance the generalization capabilities of deep convolutional neural networks (DCNNs) in applications such as deformation monitoring and topographic mapping. Its main contribution is focused in the application of interferometry using SAR imagery. The DaliWS dataset [4] was introduced as a high-spatial-resolution benchmark for water body segmentation, aimed at supporting flood disaster detection. Constructed from GaoFen-3 (GF-3) SAR satellite imagery, DaliWS provides pixel-level annotations designed to evaluate state-of-the-art segmentation networks, including FCN, SegNeXt, U-Net, and DeepLabV3+. Beyond establishing baseline benchmarks, where dual-polarization data achieved F1-scores ranging from 90.19% to 92.11%, the authors studied the impact of different polarization modes on water delineation accuracy and verified the models cross-dataset generalization capabilities, providing a high-resolution target-specific dataset for the particular application of water semantic segmentation.

This study builds a dataset constitutes an enhanced version of a previously published resource [1], which is inspired in the multitemporal fusion proposed in [5], incorporating several methodological improvements to broaden its applicability and increase its representativeness. Three major modifications were introduced in this new development. First, whereas the earlier dataset was limited to imagery from the Toronto region (Canada), covering various land-cover classes such as urban environments, water bodies, and rural landscapes, the present dataset expands its scope by including randomly selected locations distributed globally. This extension ensures greater heterogeneity in geographic and land-cover characteristics. Second, whereas the prior dataset relied on imagery acquired on fixed dates, the new dataset integrates Sentinel-1 SAR images with a corresponding 2021 ESA-Worldwide land-cover map from the same scene, sampled at random locations worldwide using Sentinel-1 images available between Jan 1, 2021 and Dec 30, 2021. The exact acquisition dates vary at each location, resulting in samples distributed throughout the entire year. This approach provides a more comprehensive temporal representation of land-cover description. Finally, the multitemporal fusion strategy was considerably refined. In the previous version, only 10 temporal acquisitions were averaged, thus limiting the dataset to a single speckle level and image quality. In contrast, the present dataset incorporates multiple fusion configurations, averaging 5, 10, 15, 20, and 25 images. This methodological upgrade produces datasets with varying degrees of speckle suppression and image quality, offering a richer framework for training and Benchmarking despeckling algorithms under diverse conditions.

## 3 Data Description

The dataset is systematically organized under a primary root directory named *dataset*. This directory contains two main subfolders: *SENTINEL1*, which stores the radar imagery, and *LANDCOVER*, which includes the corresponding land-cover reference maps obtained from the *ESA WorldCover v200* product. In addition, a CSV file is provided at the root level of the *dataset* directory, containing the complete metadata for all samples. This file includes, among other attributes, the central geographic coordinates of each image and the corresponding image identifier used in the Google Earth Engine (GEE) repository.

Within the SENTINEL1 directory, the data are further structured according to orbit direction into ASCENDING and DESCENDING subfolders. Each orbit-specific folder is subdivided into VV and VH directories, corresponding to the respective polarization channels. Individual radar images are named using a five-character random string, followed by “_n” denoting the acquisition sequence number in 2021. The name is then defined by characters in Σ∈{a,b,…,z,0,1,…,9}. The label $L_{i,n}$ defined in Eq. (1), is:

(1) $$L_{i,n}=s_{i}\;⊕n,$$

where:

− $s_{i}\in \Sigma^{k}$ is a randomly generated string of length k that uniquely identifies the scene or target area i. For this case k=5.
− n∈{1,2,…,N} represents the sequence index corresponding to the chronological order of the satellite acquisitions at different timestamps.
− ⊕ denotes the string concatenation operator.

**Fig. 1:**
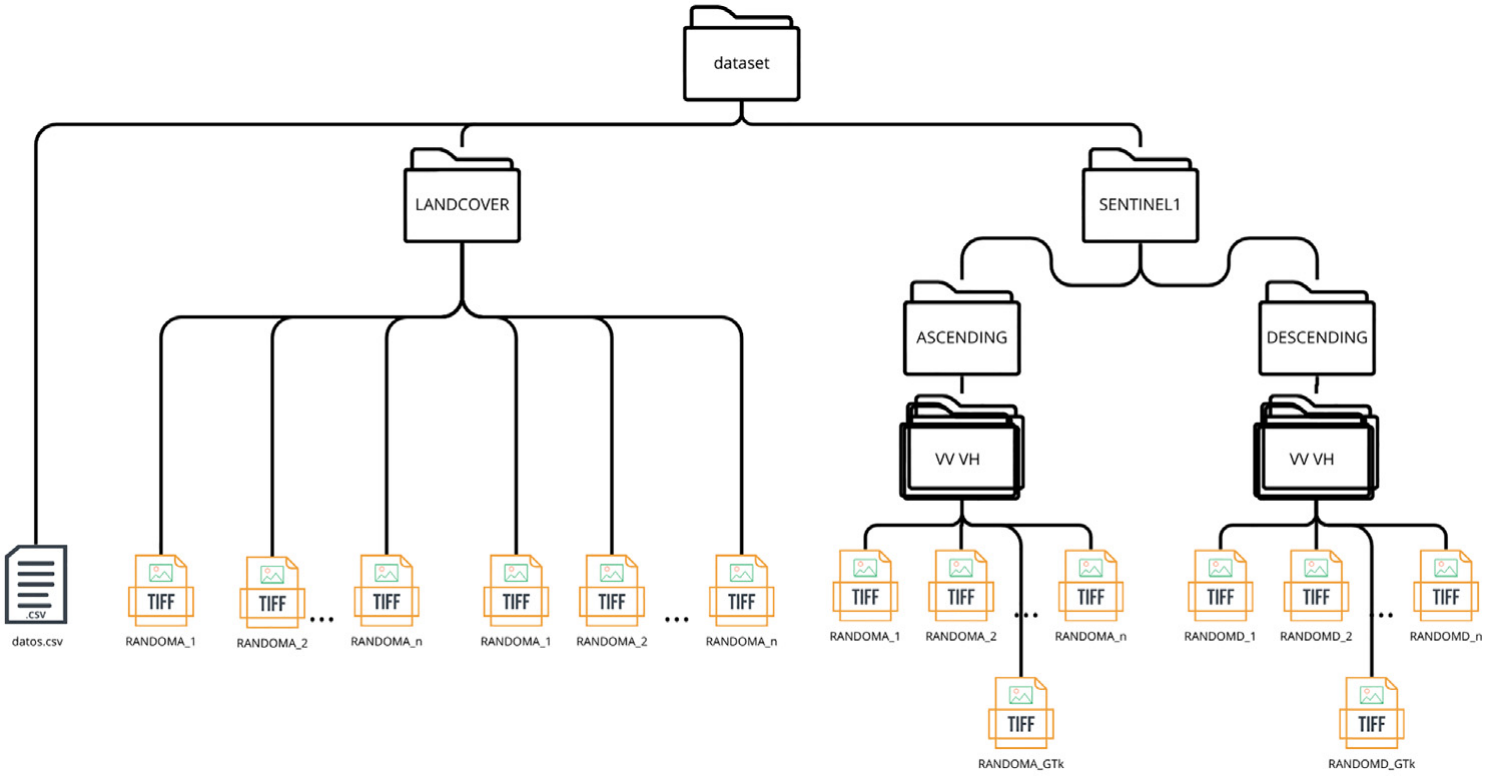
Structure of the dataset directory.

The same identifier is also used within the LANDCOVER directory to link each radar image set with the corresponding land-cover map of the region. The organization of the dataset is illustrated in Fig. 1.

**Fig. 2:**
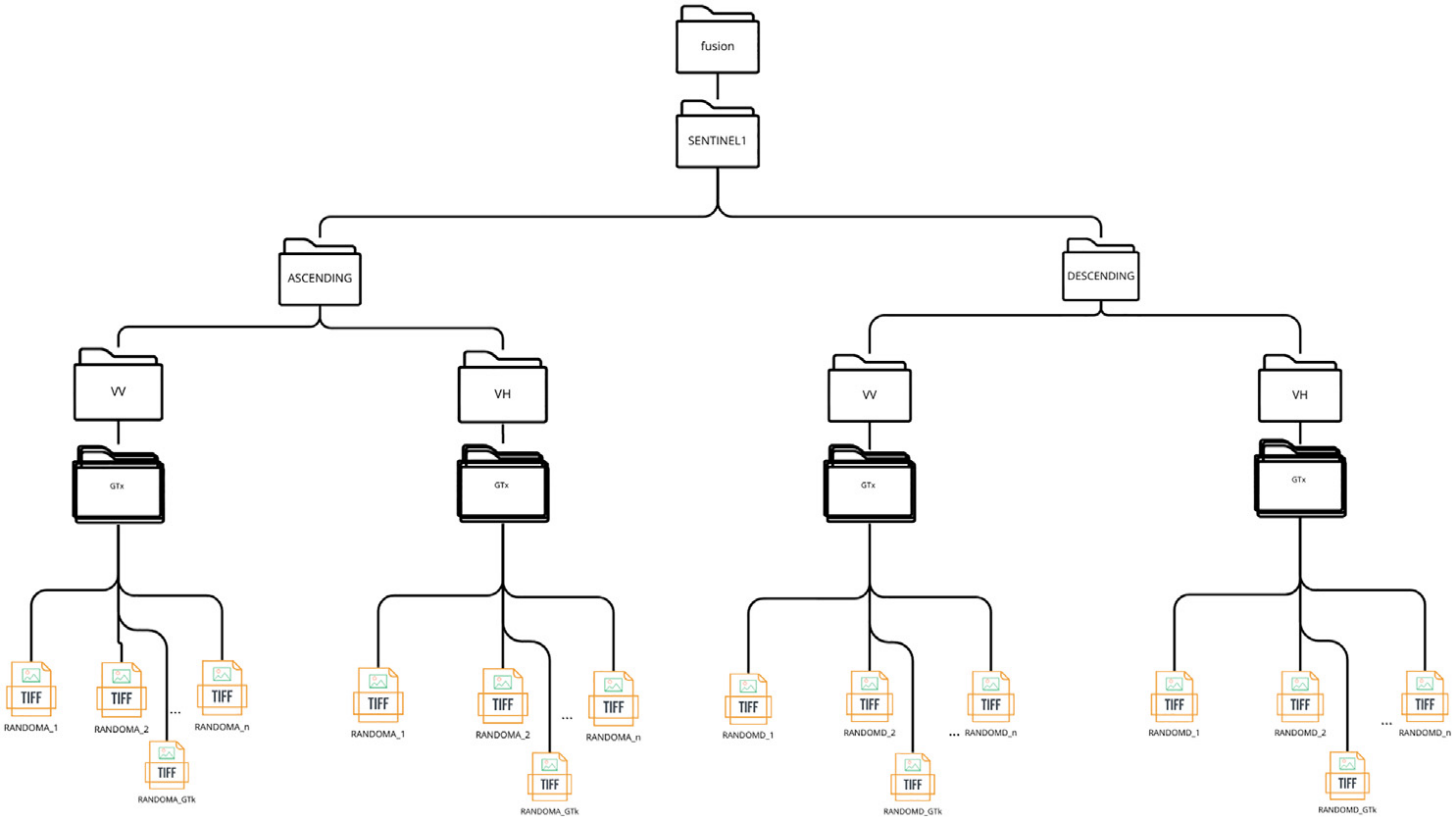
Structure of the fusion directory.

The fusion directory contains the multitemporal reference images generated from the original Sentinel-1 SAR acquisitions. As illustrated in Fig. 2, the data are first organized by satellite orbit direction (ascending and descending) and subsequently by polarization channel (VV and VH). Within each polarization folder, a set of subdirectories labeled $GT_{x}$ is provided, each containing the corresponding speckle-reduced images obtained via multitemporal fusion. In this notation, the value x denotes the number of SAR acquisitions used for temporal averaging, specifically 5, 10, 15, 20, and 25 images. Each $GT_{x}$ folder stores the fused reference images derived from the same geographic locations as the original SAR image, enabling a progressive representation of speckle attenuation as the number of combined observations increases. This hierarchical structure facilitates direct pairing between the original SAR images and their corresponding multitemporal references.

The modular architecture of the directory structure inherently supports flexible, targeted data access, enabling researchers and developers to optimize their workflows based on specific experimental requirements. Rather than using the entire dataset, this organization permits the selective extraction of data subsets. Users can effectively use subsets of interest to align precisely with the objectives of their study, for instance, using only one orbital acquisition geometry (flight direction), specific polarization channels, or different multitemporal fusion levels.

This flexibility is particularly advantageous when focusing on distinct remote sensing challenges that require varying levels of information density. For instance, in hydrological applications such as water body delineation, the analysis may be sufficiently robust using a single polarization channel, given the distinct specular backscattering characteristics of water surfaces [2]. Conversely, more complex tasks, such as land-cover classification or monitoring of biophysical vegetation parameters, typically require combining VH and VV polarizations to effectively discriminate between surface roughness and land-cover types [3].

## 4 Experimental Design, Materials, and Methods

### 4.1 Synthetic aperture radar imagery and speckle noise

SAR systems acquire microwave backscatter information from the Earth's surface using coherent imaging principles. Unlike optical sensors, SAR instruments actively transmit electromagnetic pulses and record the returned signal, enabling image acquisition independent of daylight and atmospheric conditions, such as cloud cover. This capability makes SAR particularly valuable for continuous Earth observation, environmental monitoring, disaster assessment, and land surface analysis [[4], [5], [6]].

However, this type of image is inherently speckled, a granular interference pattern arising from the coherent summation of scattered electromagnetic waves within each resolution cell. Speckle manifests as strong local intensity fluctuations that degrade visual interpretability and complicate subsequent analysis tasks, including classification, segmentation, and change detection. From a statistical perspective, speckle is commonly modeled as a multiplicative noise process, meaning that the observed SAR intensity can be interpreted as the product of the underlying reflectivity and a stochastic noise component. Because of its multiplicative nature, speckle reduction remains a challenging problem, particularly when the goal is to suppress noise while preserving structural details such as edges and fine spatial features [[7], [8], [9]].

### 4.2 Multitemporal fusion for speckle reduction

To mitigate speckle noise, an effective strategy is to leverage multitemporal SAR observations acquired over the same geographic location. When several independent SAR images of the same scene are available, temporal averaging can reduce speckle variance while preserving the underlying backscatter information. In practice, combining multiple acquisitions increases the equivalent number of looks (ENL) of the resulting image, resulting in smoother radiometric behavior in homogeneous regions and improved interpretability [10,11].

Multitemporal fusion is particularly suitable for constructing reference images for despeckling research. Since the true reflectivity of the observed surface is not directly measurable, averaging several temporally independent observations provides a practical approximation of a speckle-reduced representation of the scene. As the number of fused images increases, the speckle variance decreases approximately in inverse proportion to the number of observations. However, this process may also introduce slight smoothing effects in highly textured areas. For this reason, multitemporal averaging has been widely adopted as a strategy to generate ground-truth estimates for the development and evaluation of despeckling algorithms [12,13].

### 4.3 The role of labeled datasets in SAR despeckling research

Recent advances in machine learning and deep learning have significantly influenced the development of despeckling methods for SAR imagery. Convolutional neural networks, autoencoders, and generative adversarial networks have demonstrated promising performance in suppressing speckle while preserving structural features. These approaches, however, rely heavily on the availability of paired datasets, where noisy SAR images are associated with reference images that represent the desired speckle-reduced output [14,15].

Obtaining such labeled data remains challenging in SAR imaging because an exact ground truth without speckle is not available in real-world observations. As a result, many studies rely on simulated data or limited datasets constructed from specific geographic regions [[16], [17]]. While simulated datasets provide controlled conditions, they may not fully capture the variability of real SAR acquisitions. Conversely, real datasets derived from temporal stacks of satellite imagery can provide more realistic training conditions, including variations in land cover, acquisition geometry, and backscatter characteristics [1,12].

For this reason, the availability of diverse, well-documented datasets is essential for enabling reproducible research and unbiased benchmarking of despeckling algorithms. A dataset that includes multiple geographic regions, diverse land-cover types, and multiple levels of multitemporal fusion can serve as a valuable resource for evaluating the robustness and generalization capabilities of machine learning models [[18], [19], [20]].

### 4.4 Dataset design and generation workflow

To mitigate sampling bias and enhance the dataset's representativeness, an automated, stochastic data acquisition pipeline was implemented. The pipeline begins with the random selection of a geographic point over the global land surface. For each selected location, a rectangular region of 512×512 pixels centered on the point is extracted from Sentinel-1 imagery, considering either ascending or descending orbit directions.

For quality control, the mean backscatter value for the Sentinel-1 VH polarization is computed for each candidate region. Samples with a mean VH value lower than −20 dB are discarded, as such values are typically associated with low-information or noise-dominated areas (e.g., open-water bodies). When a sample meets this criterion, it is retained and added to the dataset.

A unique identifier consisting of a five-character random string is assigned to each accepted sample. Using this identifier, the corresponding Sentinel-1 SAR images and the associated land-cover reference map derived from *ESA WorldCover v200* are stored in their respective directories following the dataset structure described in the Data Description section. Ground-truth products are subsequently generated by temporally averaging multiple SAR acquisitions.

**Fig. 3:**
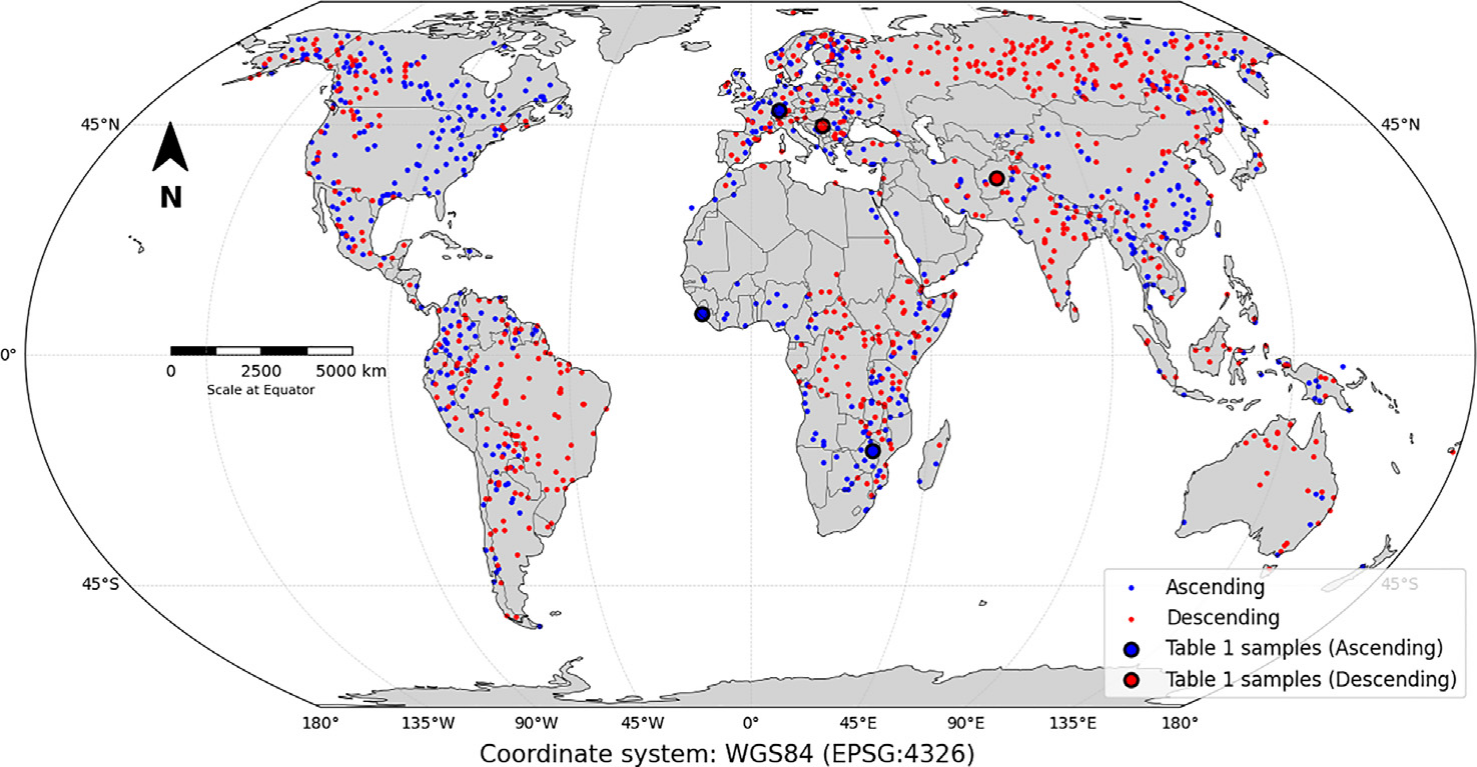
Geographical distribution of the sampled Sentinel-1 acquisition locations and orbit directions.

The entire acquisition and filtering process was implemented as a Python-based script executed in Google Colab, employing the Google Earth Engine (GEE) cloud computing platform for large-scale geospatial data access and processing. This automated framework enables scalable, reproducible, and geographically diverse sampling, thereby increasing the dataset's heterogeneity across land-cover types, climatic zones, and acquisition geometries. As shown in Fig. 3, the randomized sampling strategy yielded a geographically diverse sample set spanning multiple continents and climatic regions. Both ascending and descending orbit acquisitions are represented, ensuring variability in imaging geometry and acquisition conditions. The figure also highlights the representative samples presented in Table 1, which were selected from different geographic regions and acquisition directions.

**Table 1:** Selected samples, including filename, coordinates, location, and SAR image.

FILE NAME	COORDINATES	LOCATION	COVERAGE	DIRECTION	POLARI-ZATION
2YHtS.tiff	7°37′17.24″N, 12°18′13.35″’W	Matekan (Kenya, Africa)	Water body, vegetation	Ascending	VV
6DjK8.tiff	48°21′49.03″N, 8°27′33.23″E	Trollenberg (Germany, Europe)	Forest, crop, urban	Ascending	VV
gWUkr.tiff	17°56′50.85″S, 30°58′17.88″E	Harare (Zimbabwe, Africa)	Urban, water bodies	Ascending	VH
3OZBy.tiff	44°52′14.51″N, 20°35′35.78″E	Belgrade (Serbia, Europe)	River, Urban, vegetation	Descending	VH
7Ah2Q.tiff	33°42′56.34″N, 66°26′12.65″E	Pay Kotal (Afghanistan, Asia)	Desert, Mountains	Descending	VV

### 4.5 Samples of the dataset

**Fig. 4:**
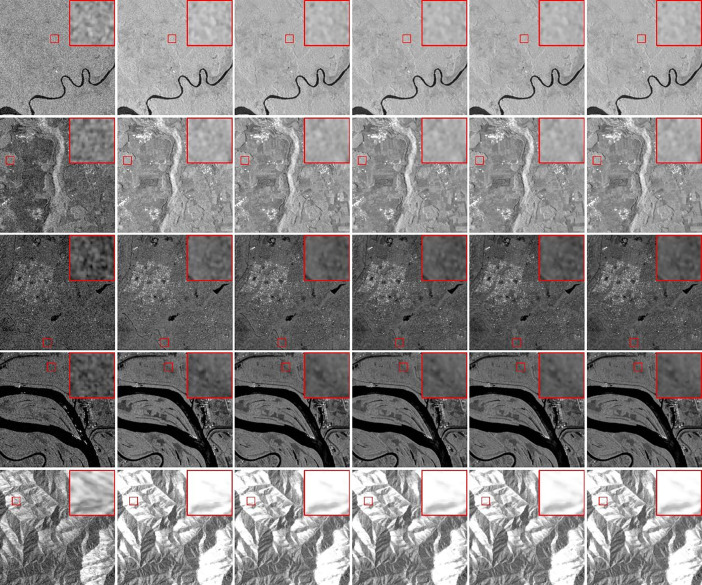
Samples of SAR images and the generated ground truth results (From top to bottom: five different samples of the dataset according to Table 1. From left to right: SAR, and multitemporal fusions with 5, 10, 15, 20, and 25 acquisitions of the same scene).

From the dataset generated, five random samples were extracted to assess quality and to inform future analyses that researchers may replicate. The description of the selected samples, including their technical specifications and resulting images, is presented in Table 1 and Fig. 4, respectively.

Fig. 4 presents five representative samples from the proposed dataset, illustrating the effect of multitemporal fusion on speckle attenuation in Sentinel-1 SAR imagery. The first column shows the original single-acquisition SAR images, and the subsequent columns present the reference images generated by multitemporal fusion using 5, 10, 15, 20, and 25 acquisitions, respectively. A progressive reduction in speckle is observed as the number of fused images increases, particularly in the homogeneous regions highlighted by the bounding boxes. At higher fusion levels, the images exhibit smoother textures, improved radiometric consistency, and enhanced interpretability, while preserving the scene's main structural elements. Although a slight reduction in local contrast may appear in highly textured regions, linear features and edges remain clearly distinguishable. These visual trends support the use of multitemporal fusion as an effective strategy for generating reliable speckle-reduced reference images, which are quantitatively assessed in the validation metrics presented in the following section.

### 4.6 Validation

To establish a reference for further training of despeckling filters, the authors calculated several well-known metrics to provide researchers and developers with a basis for assessing the quality of despeckling filters trained on this dataset. The selected metrics are the Equivalent Number of Looks (ENL) which measures the level of noise present in an homogeneous region of the images; the Mean Squared Error (MSE) which calculates the difference, pixel by pixel, of two images, in this case the different calculated ground truths with respect to the SAR image; the Structural Similarity Index (SSIM) which measures the global similarity between two images, in this case also the different ground truths with respect to the SAR image; and finally, the Peak Signal to Noise Ratio (PSNR) calculates the similarity between the ground truths and the SAR image in decibels. A more detailed explanation of these calculations and their application to SAR imagery is provided in [12]. The results of calculating these measurements on this dataset are shown in Table 2, Table 3, Table 4, Table 5.

**Table 2:** Calculation of the ENL for the different ground truths (geographic locations and coordinates correspond to the metadata established in Table 1).

Image Name	SAR	GT5	GT10	GT15	GT20	GT25
2YHtS.tif	95.87	288.68	328.52	427.73	405.54	420.04
3OZBy.tif	32.07	88.68	118.14	137.87	138.34	147.02
6DjK8.tif	72.08	197.34	296.40	326.05	326.24	306.75
7Ah2Q.tif	88.61	656,001.02	138,959.16	122,673.64	23,640.80	79,981.33
gWUkr.tif	25.32	24.84	43.01	38.62	39.36	41.65

**Table 3:** Calculation of the MSE for the different ground truths (geographic locations and coordinates correspond to the metadata established in Table 1).

Image Name	GT5	GT10	GT15	GT20	GT25
2YHtS.tif	1113.27	865.50	861.98	834.55	892.81
3OZBy.tif	527.04	428.63	407.71	400.59	397.41
6DjK8.tif	3127.30	2737.46	2596.07	2681.96	2629.96
7Ah2Q.tif	2049.76	2112.53	2011.45	1976.42	2063.49
gWUkr.tif	872.39	561.83	455.70	416.22	424.98

**Table 4:** Calculation of the SSIM for the different ground truths (geographic locations and coordinates correspond to the metadata established in Table 1).

Image Name	GT5	GT10	GT15	GT20	GT25
2YHtS.tif	0.56	0.59	0.59	0.60	0.60
3OZBy.tif	0.62	0.64	0.64	0.65	0.65
6DjK8.tif	0.52	0.53	0.55	0.55	0.55
7Ah2Q.tif	0.63	0.63	0.63	0.64	0.64
gWUkr.tif	0.56	0.58	0.59	0.60	0.60

**Table 5:** Calculation of the PSNR for the different ground truths (geographic locations and coordinates correspond to the metadata established in Table 1).

Image Name	GT5	GT10	GT15	GT20	GT25
2YHtS.tif	17.66	18.76	18.78	18.92	18.62
3OZBy.tif	20.91	21.81	22.03	22.10	22.14
6DjK8.tif	13.18	13.76	13.99	13.85	13.93
7Ah2Q.tif	15.01	14.88	15.10	15.17	14.98
gWUkr.tif	18.72	20.63	21.54	21.94	21.85

## Limitations

• The methodology used to construct this dataset is highly adaptable and can be readily replicated using data from different geographic regions or alternative SAR sensors. This flexibility enables the generation of substantially larger datasets, which are essential for training more sophisticated and computationally demanding deep learning models. However, the generation of reference targets via multitemporal averaging relies on the assumption of surface stationarity over the acquisition window. In regions experiencing rapid land-cover transitions, such as seasonal agricultural dynamics, urban expansion, or episodic flooding events, temporal decorrelation may introduce blurring artifacts or artificial smoothing in the averaged ground-truth images, which can be more at higher fusion levels.
• The procedure is structured as a sequence of well-defined steps. First, actual SAR images corresponding to a randomly selected geographic area are downloaded. These images are subsequently rescaled to a standardized dynamic range of 0–255 to facilitate uniform preprocessing. Next, image registration is performed with respect to a designated reference acquisition, ensuring spatial alignment and consistency across the dataset. Following this, a multitemporal fusion step is applied, integrating information from multiple temporal acquisitions to enhance the quality and reliability of the ground-truth representation.
• The radiometric and geometric characteristics of the dataset are strictly tied to the Sentinel-1 and its specific spatial resolution constraints. Deep learning models trained exclusively on this dataset may require fine-tuning or domain adaptation before being deployed on very-high-resolution commercial SAR imagery Tables 3,4.
• The benchmarks in Table 2, Table 3, Table 4, Table 5 provide a standardized baseline for evaluating new despeckling models, focusing strictly on classical full-reference structural and radiometric criteria. To extend this evaluation, future studies could incorporate no-reference metrics or learning-based perceptual quality indicators (such as NIQE, BRISQUE, or LPIPS also used in the medical context [20]) to better align quantitative performance with human visual perception.

## Ethics Statement

The authors confirm that, after reading the ethical requirements for publication in Data in Brief, the current work does not involve human subjects, animal experiments, or any data collected from social media platforms.

## CRediT Author Statement

**Jean Pierre Díaz-Paz:** Conceptualization, Software, Investigation, Resources, Writing - Original Draft; **Ahmed Alejandro Cardona-Mesa:** Conceptualization, Methodology, Writing - Original Draft; **Paula Andrea Muñoz-Uribe:** Investigation, Resources, Writing - Original Draft; **Rubén Darío Vásquez-Salazar:** Conceptualization, Methodology, Writing - Original Draft; **Santiago Zapata-Vargas:** Investigation, Resources, Writing - Original Draft.

## Data Availability

Mendeley DataLabeled Dataset of SAR Sentinel-1 Imagery Despeckled with Multitemporal Fusion (5–25 Looks, VV/VH Polarizations) (Original data).
